# Behavior change techniques in physical activity and dietary interventions among adolescent and young adult cancer survivors: a systematic review and meta-analysis of randomized controlled trials

**DOI:** 10.1007/s11764-025-01836-y

**Published:** 2025-06-27

**Authors:** Erin M. Coffman, Susanna M. Choi, Andrew B. Smitherman, Erik A. Willis, Stephanie L. Martin, Deborah F. Tate, Carmina G. Valle

**Affiliations:** 1 Department of Nutrition, Gillings School of Global Public Health, University of North Carolina at Chapel Hill, Chapel Hill, NC USA; 2 Lineberger Comprehensive Cancer Center, University of North Carolina at Chapel Hill, Chapel Hill, NC USA; 3 School of Medicine, University of North Carolina at Chapel Hill, Chapel Hill, NC USA; 4 Center for Health Promotion and Disease Prevention, University of North Carolina at Chapel Hill, Chapel Hill, NC USA; 5 Present Address: Department of Implementation Science, Wake Forest University School of Medicine, Winston Salem, NC USA

**Keywords:** Survivors, Adolescent, Young adult, Neoplasms, Diet, Exercise

## Abstract

**Purpose:**

This systematic review and meta-analysis aimed to synthesize the evidence from physical activity (PA) and dietary behavior change interventions designed specifically for adolescent and young adult (AYAs) cancer survivors. We identified study characteristics and behavior change techniques (BCTs), examined intervention effectiveness, and explored study characteristics and BCTs associated with effectiveness.

**Methods:**

A comprehensive search of multiple databases was conducted (March 2024) for randomized controlled trials (RCTs) conducted among post-treatment cancer survivors diagnosed during adolescence or young adulthood (15–39 years), comparing PA or dietary outcomes. Two reviewers screened all studies, extracted data, and characterized interventions by BCTs. Analyses weighted effect sizes (Hedges *g*) by their inverse variance and combine them using multilevel random effects meta-analytic procedures. Random effects univariate regressions examined the association between study characteristics, BCTs, and effectiveness.

**Results:**

Fourteen RCTs (*n* = 1169) were included for review. All studies had multiple outcomes of interest, including PA; two included dietary components. There was a significant overall intervention effect on PA (0.378, 95% CI, 0.183–0.573; *p* = 0.002). Significant moderators were the type of PA outcome assessed, the use of two behavioral theories, and biofeedback. Other BCTs showed notable differences in effect size, though not significant.

**Discussion:**

Interventions improved PA outcomes in AYA cancer survivors. Most studies were pilot or feasibility trials, lasting three months. Future research should prospectively identify BCTs and examine their effects post-intervention. Interventions targeting both PA and dietary behaviors are lacking.

**Implications for Cancer Survivors:**

There is a need for behavioral interventions designed specifically to meet the unique needs of AYA cancer survivors and future studies should include PA and dietary components, larger and more diverse samples, and longer follow-up periods.

**Registration:** PROSPERO (reference: CRD42022198889).

## Introduction

Each year in the USA, around 85,000 adolescents and young adults (AYAs), ages 15–39, are newly diagnosed with cancer [1]. Due to improvements in detection, treatment, and supportive care, the 5-year relative survival rate for AYAs exceeds 85%, resulting in a growing population of over 2 million AYA survivors [1, 2]. However, as a result of their disease and treatments, survivors of cancer during adolescence and young adulthood are at increased risk for long-term and late effects on all major organ systems, which can lead to chronic health conditions like cardiovascular risk factors and disease, obesity, diabetes, physical and mental impairments, as well as cancer recurrence or secondary cancers [3–9]. These chronic health conditions are associated with poor health-related quality of life (HRQoL), frailty, and premature mortality [7, 10].

Adverse health outcomes are exacerbated by modifiable lifestyle factors such as low physical activity (PA), sedentary lifestyles, and poor diet quality [11]. While the American Cancer Society (ACS) recommendations for cancer survivors include achieving and maintaining a healthy body weight, being physically active, and consuming a healthy diet [12, 13], the majority of AYA cancer survivors are not adherent to them [14, 15]. Less than half (46%) of AYAs meet recommendations for vegetable consumption, 67% for fruit consumption, and less than half meet recommendations for aerobic or resistance PA (31% and 47%, respectively) [14].

Most research on PA and dietary interventions has been conducted in adult cancer populations; few empirical studies have investigated PA interventions in AYAs specifically, and even fewer have examined dietary behaviors or outcomes. In 2016, Pugh et al. conducted the first systematic review of health behavior change interventions among AYA survivors, synthesizing 12 studies published between 2009 and 2014, which focused on a range of health behaviors, including PA, diet, and tobacco use [16]. Due to the heterogeneity of study designs, the authors chose not to conduct a meta-analysis but identified trends in the health behavior interventions specific to AYA survivors. Common components used included remote intervention delivery, the provision of social support, personalized risk counseling regarding potential late effects of treatment, and involving AYA survivors in the decision process [16].

More recent reviews have explored behavioral interventions and their impacts on health outcomes for AYAs across the cancer continuum [17–20]. These reviews found that PA interventions are feasible, acceptable, and safe for AYAs during cancer treatment and beyond and positively affect health-related outcomes despite heterogeneity in study designs, sample characteristics, and outcomes studied [21]. They also highlight the gaps in the design and implementation of health behavior interventions for AYAs, and the need to identify optimal characteristics of interventions that align with the specific needs and preferences of AYAs, and improve survivorship outcomes [18, 19, 21]. While extensive research has shown that theory-based health behavior interventions have larger effect sizes than those without theoretical foundations [22], few of these previous reviews have examined the theoretical foundations of behavioral interventions for AYAs, or the relationship between specific behavior change theories, intervention strategies, and health outcomes.

To further categorize effective intervention strategies, Abraham and Michie developed a behavior change technique (BCT) taxonomy to explicitly describe interventions’ theoretical basis and active components that can strengthen the study design, implementation, analysis, and replication [23, 24]. Coding the BCTs of existing interventions and comparing efficacy across studies may help to identify successful components for future interventions. The current study expands upon previous reviews by utilizing BCTs to characterize interventions evaluated in randomized controlled trials (RCTs) among post-treatment AYA survivors and conducting a meta-regression to analyze the effect of individual BCTs on behavioral outcomes.

To address the limitations of previous reviews, the purpose of this systematic review and meta-analysis was to (1) characterize RCTs that evaluated PA and dietary behaviors in post-treatment AYA cancer survivors, (2) evaluate the efficacy of interventions on PA or dietary behavior change, (3) identify behavioral theories and BCTs that have been utilized, and (4) explore which BCTs are associated with improved outcomes in PA or dietary behavior outcomes for AYAs. Identifying the intervention components and BCTs associated with improved health behaviors can help provide insight into how behavior change interventions improve health outcomes among AYA cancer survivors and inform the development of future interventions.

## Methods

**Fig. 1:**
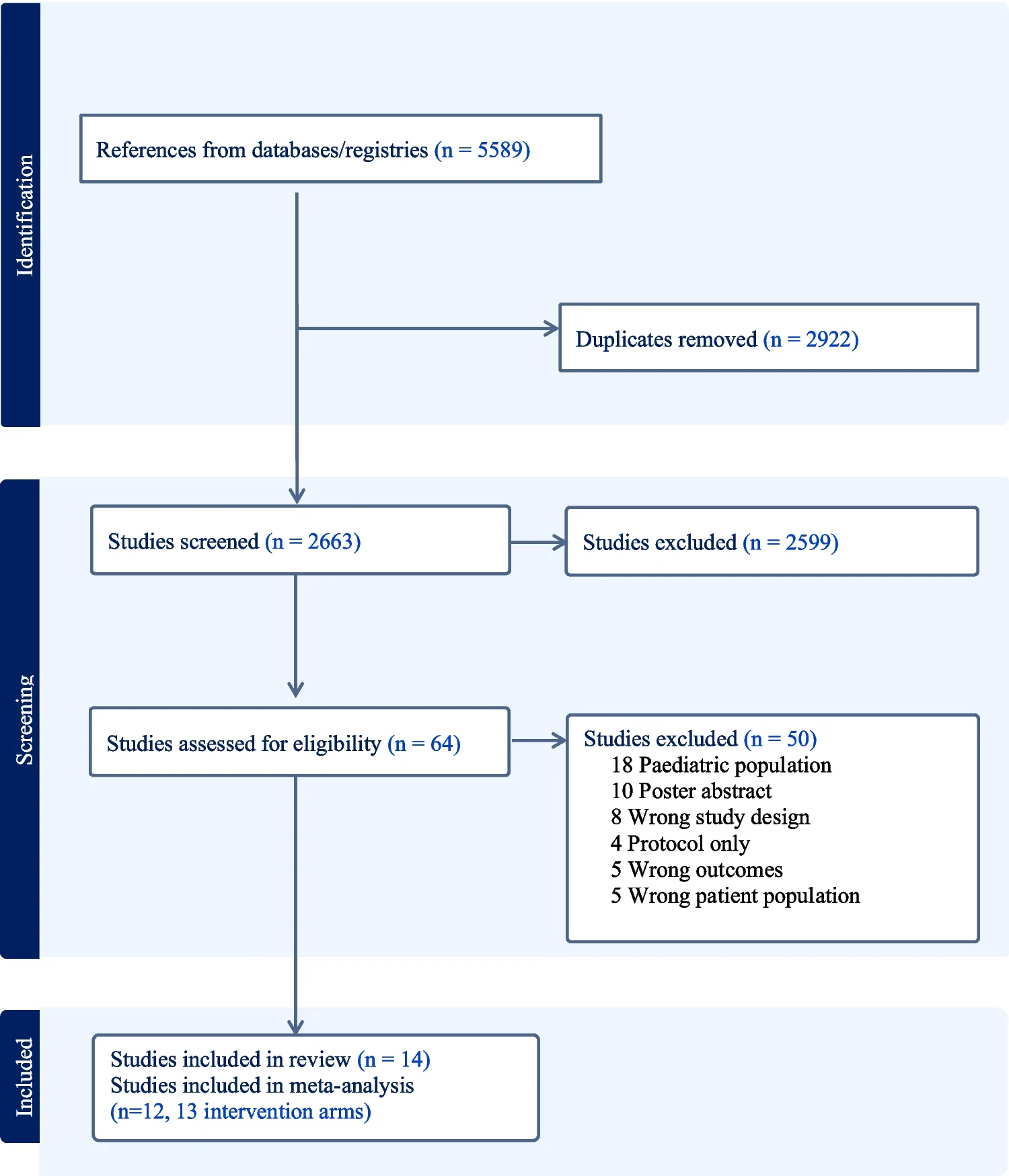
PRISMA flowchart

The review was conducted following the Preferred Reporting Items for Systematic Reviews and Meta-Analysis (PRISMA) guidelines (Fig. 1), and the protocol was pre-registered with PROSPERO (reference: CRD42022198889) [25].

### Search strategy

A comprehensive search strategy was utilized to identify relevant literature. Searches were conducted in March 2024, and all applicable studies before this date were considered for inclusion. The search strategy involved comprehensive searches of multiple databases: PubMed, EBSCO (CINAHL, MEDLINE, PsycINFO, Academica Search Premier, etc.), Web of Science, EMBASE, and Cochrane CENTRAL using keywords related to interventions (e.g., RCT, random, controlled trial), nutrition (e.g., diet, energy balance, energy intake), PA (e.g., exercise, aerobic), cancer (e.g., neoplasm, oncology), and AYAs (e.g., AYA, teenager, adolescent, young adult). Boolean strings were used to combine keywords with “AND” and “OR” operators to maximize relevant literature. A complete list of the search strings can be found in the Appendix. Next, reference lists of the reviews and articles captured by the literature search were examined for relevant studies not identified by the search. All grey literature obtained during the search was considered, but none was included. Clinicaltrials.gov was cross-checked for potential literature in progress or under reviews, though no additional trials were eligible for inclusion at the time of the search.

### Study selection, inclusion, exclusion

All identified literature was screened for relevance. To be included, articles needed to meet the following inclusion criteria based on the PICO framework [26]: used a randomized controlled design, diagnosed with cancer of any type, completed active treatment, participants were mean age ≥ 15 and ≤ 39 years of age at cancer diagnosis, and included interventions with a primary (or secondary) outcome measure of PA or diet. For PA interventions, studies were only included if PA was measured at baseline and immediately after the intervention. Nutrition interventions had to include a manipulated nutritional component and measure a change in outcome from baseline to post-intervention. Studies were excluded if they were pharmacologic interventions only, if dietary prescription and/or exercise prescription did not include additional counseling targeting psycho-behavioral change, studies of herbal supplements, vitamins, and minerals, and if they only included patients at risk of weight loss (e.g., wasting disease, eating disorders, head and neck cancers). Studies across all contexts and settings that met the criteria were included. Comparators included non-exposed control, alternative intervention, education or self-help control, or delayed intervention control groups. Only RCTs with reports written in English were included.

The initial database searches yielded 5589 potential references. After removing duplicates, there were 2663 references. We managed the review process using Covidence Online software (https://www.covidence.org). Two reviewers (EMC, SMC) independently examined all study titles for relevance, excluding 2599 references. Abstracts were reviewed for the remaining 79 studies, and 65 relevant articles were identified for full-text screening. All conflicts were reviewed and resolved through group discussion. Finally, the two reviewers (EMC, SMC) independently examined the 65 full-text articles for inclusion and tracked reasons for exclusion, resolving conflicts through group discussions. From the full-text review of 64 articles, 23 studies (36%) were excluded because they did not focus on AYA cancer survivors, 21 studies (33%) were not RCTs with a focus on PA or dietary behaviors, 13 were poster abstracts only, and three were protocol papers. Ultimately, 15 studies were included for systematic review, and 13 studies representing 14 intervention groups were included in the meta-analysis.

### Data extraction and article coding

To characterize RCTs that evaluated PA and dietary behaviors in post-treatment AYA cancer survivors, two independent reviewers (EMC, SMC) extracted data using standardized spreadsheets and Covidence Online software, resolving conflicts through discussion. Two independent reviewers (EMC, SMC) extracted data using standardized spreadsheets, resolving disputes through discussion. Extracted features included *basic study information* such as title, publication year, authors, journal name, country of origin, and *study characteristics* such as study design and program description, theoretical basis, delivery mode, setting, profession of interventionists, length of intervention, follow-up, retention rates, and outcomes assessed. All outcomes reported were collected. *Participant characteristics* included age, gender, race/ethnicity, and body mass index (BMI). *Methodological* features included characteristics of the intervention and control conditions and aspects of study quality and rigor, which were assessed using the Cochrane Collaboration’s Tool for Assessing Risk of Bias [27].

### Behavior change techniques

Intervention and control groups of each study included in the systematic review (*N* = 14, 15 intervention groups) were coded for BCTs using the BCTTv1 taxonomy as present versus absent (1 and 0, respectively) for all intervention and control conditions by two trained coders (EMC, SMC) [28]. All studies were coded by EMC, who has extensive experience coding BCTs, including a published review [29]. A second coder (SMC) independently coded a subset of studies (*N* = 5, 35%), and any discrepancies were resolved through discussion. BCTs were coded only when their use was explicitly described in the text with unambiguous evidence of their application to the targeted behavior and population. Complete protocols, related papers, and supplementary materials were utilized when available. In the case of multiple intervention arms, BCTs and outcome data were extracted for each intervention arm and compared to the control condition.

To evaluate the potential efficacy of BCTs identified in interventions, a “promise ratio” was used to characterize the impact of individual BCTs on improving participants’ health behaviors. This method was developed by Gardner et al. [30] and has been used in systematic reviews and meta-analyses of PA interventions for breast and prostate cancer survivors [31–33]. Each study was characterized according to its potential to improve health behavior outcomes post-intervention. Studies were coded as “very promising” if there were statistically significant differences in PA or dietary outcomes between the intervention and control groups post-intervention (between-group differences), “quite promising” if there were significant differences from baseline to post-intervention in the intervention group and more than the control group (within-group differences), and “non-promising” if studies did not report statistically significant differences between groups or from baseline to post-intervention. Next, each BCT was given a “promise ratio” based on the times it was utilized in promising vs non-promising studies. Promise ratios were not calculated for BCTs only included in non-promising interventions. An individual BCT had to have a promise ratio of ≥ 2 to be considered promising, with higher ratios indicating a more promising intervention. Promising BCTs identified in intervention groups and their association with change in PA effect sizes were explored further in moderation analyses.

### Risk of bias

Study quality and rigor were assessed using the Cochrane Collaboration’s Tool for Assessing the Risk of Bias [27]. The tool identifies five sources of bias: bias arising from the randomization process, bias due to deviations from the intended intervention, bias due to missing outcome data, bias in measuring the outcome, and bias in selecting the reported outcome.

### Meta-analytic approach

All analyses were conducted using the *Metafor* and *clubSandwich* packages for R (version 4.3.1). To evaluate the efficacy of interventions on PA, we characterized effect sizes for each intervention using the standardized mean difference (SMD), the difference in intervention and control means divided by the pooled standard deviation, with a correction for small sample sizes, Hedges’ *g* [34]. Effect sizes for PA interventions represent the difference in the amount of PA from baseline to follow-up for the intervention group compared to the control group for each PA outcome reported (i.e., minutes per week of moderate-vigorous PA (MVPA), light PA, total PA, sedentary behavior, walking, steps/day, and VO^2peak^). Where moderate and vigorous PA were reported separately, their means were added together, and the SD was calculated for the MVPA amount. Each intervention group was compared to the control group for studies with two or more intervention groups. If data were reported for multiple time points, data reported from the follow-up immediately post-intervention was used to calculate effect sizes. Effect sizes were interpreted according to Cohen’s conventions as small (0.2–0.49), moderate (0.5–0.79), and large (≥ 0.8) [35]. Effect sizes were calculated using the data reported in the articles (i.e., means and standard deviations or standard errors, change scores) [34].

The mean effect size across studies was calculated using a three-level model with robust variance estimations (RVE), which provides a way to include dependent effect sizes nested within studies with unbiased coefficient estimates and valid standard errors [36]. Applying the three-level meta-analysis allows for an overall pooled effect size estimate while accounting for sampling variance (level 1), variance between effect sizes extracted from the same study (level 2), and variance between studies (level 3) [37]. Meta-regression coefficients were estimated using random effects models with RVE, which uses restricted maximum likelihood (REML), with an adjustment to reduce small sample bias [36, 38, 39].

Heterogeneity across studies was evaluated using the *Q*-statistic and *I*^2^ (the percentage of total variation between studies). We followed Cochrane Handbook recommendations to interpret I^2^ values as small (≤ 0.39), moderate (0.4–0.69), and large (≥ 0.8). [27] A significant *Q*-value indicates heterogeneity and warrants examination of moderator variables. Next, we checked for publication bias using funnel plots and Egger’s regression. Duval and Tweedie’s [40] (2000) trim-and-fill procedure was used to correct for publication bias.

### Moderator analyses

We explored heterogeneity of the overall pooled effect size by extending the three-level model to include the a priori identified moderators in univariate meta-regression models. We examined whether any intervention characteristics were associated with intervention effectiveness: PA outcome measured, type of control group, objective vs. self-report outcome measurements, theoretical basis and theoretical theories used, the total number of BCTs utilized in intervention groups, and individual BCTs present. Only BCTs with a promising ratio of ≥ 2 were used to avoid inflation of the results [41].

## Results

Table 1 summarizes the characteristics of the fourteen studies included in the systematic review. All studies were published between 2005 and 2024, and the majority were conducted in the USA (*N* = 8, 60%) [42–49], with others in Canada (*N* = 2) [50, 51], Australia (*N* = 1) [52], Germany (*N* = 1) [53], Norway (*N* = 1) [54], and China (*N* = 1) [55]. In total, 1169 participants were included in the fourteen studies. Study sample sizes ranged from 16 to 280 (median, 56 participants), with 8 to 140 participants per intervention arm (median, 30 participants) [50]. All studies included AYA cancer survivors, with a mean age of 29.2 (SD 5.5, range 15–48). Participants were primarily female (64%) and non-Hispanic White (80%). Mean BMI ranged from 25 to 33.4 kg/m^2^ (median, 29.8 kg/m^2^). Studies included post-treatment survivors of various cancer types, including both hematopoietic malignancies and solid tumors.

**Table 1:** Characteristics of Lifestyle Interventions for AYAs (*N* = 14)

First author (year), country, purpose	Population	Intervention components, duration, theory, BCTs	Outcome measures	Main findings
**Atkinson** [52] (2020), Australia To assess the effects of a 10-week structured exercise intervention on cardiorespiratory fitness compared to controls in AYAs who had recently completed acute system cancer treatment	43 AYAs age 15–25 at diagnosis, completed treatment within previous 2 months I: exercise group (*n* = 22) C: standard care (*n* = 21) Age (m ± SD): 20 ± 3 Gender: 47% female Race/ethnicity: NR BMI (m ± SD): 25(6)	In-person 10-week aerobic and resistance supervised exercise training intervention 2 sessions per week with accredited exercise physiologists Assessments at baseline, 10 weeks, and 6 months	VO_2peak_ Strength and flexibility Leisure score index (LSI) BMI QOL Fatigue Adherence	Theory: NR BCTs: I: 16 C: 0 • Exercise adherence was 90% • Retention was 91% at 10 weeks and 78% at 6 months • Promising rating: very promising
**Bélanger** [50] (2014), Canada To explore the effects of targeted PA print material on PA and QoL in young adult cancer survivors (YACS)	212 YACS age 18–39 at diagnosis, within 5 years of diagnosis I: tailored PA guidebook (TPAG) (*n* = 106) C: Canadian PA Guidelines (CPAG) (*n* = 106) Age: 18–29 (25.9%) 30–39 (74.1%) Gender: 60.8% female Race/ethnicity: 85.4% Non-Hispanic White BMI: < 25 (50.5%) ≥ 25 to < 30 (30.7%) ≥ 30 (18.9%)	Remote Mailed materials 12 weeks Targeted PA guidebook (TPAG) compared to generic Canadian PA guidelines (CPAG) Assessments at baseline, 1 month, 3 months	Total PA Vigorous PA Moderate PA QoL Adherence	Theory: TPB BCTs: I:11 C: 8 • Retention was 67% at 1 month and 60% at 3 months • Promising rating: promising
**Johnson** [42] (2022), USA Evaluate the feasibility of a Fitbit Flex and Facebook intervention designed to promote PA among AYAS, with a focus on increasing steps	50 AYAS age 18–39, > 1 and < 6 years from diagnosis, not meeting PA guidelines I: (*n* = 26) C: Fitbit only (*n* = 23) Age (m ± SD): 33.6 (4.9) Gender: 55.1% Female Race/Ethnicity: 63.3% Non-Hispanic White BMI (m ± SD): 29.5 (7.5)	Remote mHealth 12 weeks Fitbit, Facebook group, Weekly step count goal setting, PA “buddy” system, text messages Assessments at baseline and 3 months	Feasibility MVPA Light PA Sedentary Time (ST) Physical function Fatigue	Theory: SDT BCTs: I:28 C:5 • 3-month retention was 98% • Promising rating: non-promising
**Keadle** [43] (2021), USA To evaluate the feasibility, acceptability, and preliminary efficacy of an eHealth intervention with charity-based incentives to increase PA among AYAS	51 AYAS age 18–50 diagnosed 15–39, ≥ 6 months post-treatment, reporting < 60 min MVPA/week PA (*n* = 25) PA + Charity (*n* = 26) Age (m ± SD): 36.9 (5.2) Gender: 86.2% female Race/ethnicity: 61.6% Non-Hispanic White BMI (m ± SD): 33.4 (8.3)	Remote eHealth 12 weeks PA group (Fitbit, step goal, weekly electronic newsletter) PA + charity (Fitbit, step goal, weekly electronic newsletter, charity donation if step goal achieved) Assessments at baseline and 3 months	Feasibility Acceptability Fidelity MVPA Light PA Sedentary behavior Steps MET-hrs PROs (physical function, fatigue, depression, sleep disturbance)	Theory: SCT and SDT BCTs: PA: 19 PA + C: 27 • 3-month retention was 88% • Promising rating: very promising
**Kepper** [44] (2024), USA To determine feasibility and preliminary effectiveness of a digital tool on AYAs’ PA and food intake behaviors, and cardiovascular health (CVH)	50 AYAs age 12–39, ≥ 6 months post-treatment, BMI ≥ 25 I: (*n* = 23) C: Waitlist control (*n* = 27) Age (m ± SD): 19.83 (5.2) Gender: 46% female, 50% male, 4% nonbinary/transgender Race/ethnicity: 82% non-Hispanic White BMI (m ± SD): 31.8 (5.7)	remote mHealth Counselling with health-care team using PREVENT digital health tool Using EHR and self-report patient data, algorithm generates monthly progressive tailored goals for PA and food intake Interactive resource map and repository of community resources Assessments at baseline and 3 months	Feasibility MVPA Food recommendations met (1–5) BMI CVH risk score (0–14) Willingness to change Self-efficacy Knowledge	Theory: SDT BCTs: I:20 • 3-month retention was 76% • Promising rating: very promising
**Li** [55]* (2023), China To evaluate the effects and compare the difference between the PA and behavioral activation (BA) on AYAs’ psychological distress	153 AYAs age 15–39, diagnosis < 5 years I: PA (*n* = 47) I: PA + behavioral activation (BA) (*n* = 48) C: standard care (*n* = 48) Age (m ± SD): 29.61 (6.35) Gender: 76.2% female Race/ethnicity: NR BMI (m ± SD): 32.8 (5.3)	Remote individual and group mHealth 8 weeks 3-arm parallel group **PA group received:** exercise manual, PA tracker, WeChat group including friends and family **PA + BA** group also received weekly group therapy online Assessments at baseline, 1-week post-intervention, and 3 months	Salivary cortisol and testosterone Anxiety and depression MVPA Social support Self-efficacy Sleep quality	Theory: SCT and TPB BCTs: PA: 30 BA: 33 C: 7 • Promising rating: very promising
**Rabin** [45] (2011), USA To assess intervention feasibility and acceptability. Secondary aims were to assess key outcomes (PA, mood, fatigue) to generate effect size estimates	18 AYAs age 18–39, diagnosis < 10 years, sedentary lifestyle I: *n* = 8 C: Standard Care (*n* = 10) Age (m ± SD): 32.17 (5.58) Gender: 55.56% female Race/ethnicity: 94.4% Non-Hispanic White BMI (m ± SD): 29.7 (8.6)	Remote mHealth 12 weeks PA website, online forum, weekly emails Assessments at baseline and 3 months Theory: SCT and TTM	MVPA Fatigue Mood	BCTs: I: 14 C: 0 • Promising rating: promising
**Rabin** [46] (2016), USA To determine feasibility and acceptability of the RENEW intervention, and explore whether intervention increased PA, fitness, and mood	35 AYAS, age 18–39, diagnosis < 10 years, sedentary lifestyle I: *n* = 19 C: Waitlist Control (*n* = 16) Age (m ± SD): 32.88 (4.88) Gender: 82.9% female Race/ethnicity: 74.3% Non-Hispanic White BMI: NR	Remote 12 weeks Telephone-delivered PA and meditation intervention Weekly phone calls, pedometers, online forum Assessments at baseline, 12, and 24 weeks	MVPA Aerobic fitness Mood	Theory: SCT and TTM BCTs: 28 • 77% retention at 24 weeks • Promising Rating: Very promising
**Salchow** [53] (2021), Germany To determine the effect of a counselling-based intervention with the focus on improved vigorous PA	89 AYAs age 15–39, with ≥ 1 risk factor for cardiovascular disease I: Individual counselling (*n* = 45) C: Standard Care (*n* = 44) Age (m ± SD): 24.1 (6.3) Gender: 55% female Race/ethnicity: NR BMI (m ± SD): 23.5 (5.1)	Remote 12 weeks One counselling session with sports scientists, telephone follow-up after weeks 1, 3, and 12 Assessments at baseline, 12 and 52 weeks Theory: TTM	MVPA QoL	BCTs: I: 22 C: 3 • Retention was 69% at 12 weeks and 61% at 52 weeks • Promising rating: promising
**Schwartz** [47] (2020), USA To assess the feasibility and acceptability of a text messaging intervention designed to improve physical and psychosocial well-being	61 AYAs age 12–25, < 1 year from end of treatment I: *n* = 31 C: Standard care (*n* = 30) Age (m ± SD): 17.24 (2.94) Gender: 56% female Race/ethnicity: 64.0% Non-Hispanic White BMI: NR	Remote mHealth 16 weeks 1–2 daily text messages, AYA survivorship handbook Assessments at baseline and 16 weeks	Feasibility Acceptability Health behaviors (fruit/vegetable, whole grain, and processed food intake, sunscreen use, PA, alcohol, smoking) Health knowledge Health competence beliefs HRQOL	Theory: SCT and HPM BCTs: I: 10 C: 4 • Promising rating: very promising
**Thorsen** [54] (2005), Norway To evaluate the effect of a supervised, home-based, PA training program on cardiorespiratory fitness and HRQOL	139 cancer survivors aged 18–50, completed treatment within 1 month I: *n* = 59 C: Standard Care (*n* = 52) Age (m ± SD): 39.1 (8.4) Gender: 68% female Race/ethnicity: NR BMI (m ± SD): 29.8 (7.0)	In-person Home-based, supervised by exercise instructor 14 weeks Minimum 2 sessions per week of at least 30 min Assessments at baseline and 14 weeks	VO_2max_ HRQoL Anxiety Depression	Theory: NR BCTs: I: 14 C: 0 • Retention was 80% at 14 weeks • Promising rating: very promising
**Valle** [48] (2013), USA To evaluate the feasibility and preliminary efficacy of a Facebook delivered intervention aimed at improved PA behaviors	86 AYAS ages 21–39, diagnosed at ≥ 18, ≥ 1-year post-diagnosis, not meeting PA recommendations I: *n* = 45 C: Self-help (*n* = 41) Age (m ± SD): 31.7 (5.1) Gender: 91% female Race/ethnicity: 91% Non-Hispanic White BMI (m ± SD): 28.7 (4.5)	Individualized eHealth 12 weeks Fitbit, moderated Facebook group, study website with weekly goal setting and feedback Self-help Comparison (Fitbit, unmoderated Facebook group) Assessments at baseline and 12 weeks	Feasibility MVPA Body Weight BMI QoL Adherence Acceptability	Theory: SCT BCTs: I: 28 C: 20 • Retention was 77% at 12 weeks • Promising rating: very promising
**Valle** [49] (2023), USA To evaluate the effects of a 6-month theory-based mobile PA intervention	280 AYAS ages 18–39, diagnosis < 10 years, not meeting PA recommendations I: *n* = 140 C: Self-help (*n* = 140) Age (m ± SD): 33.4 (4.8) Gender: 82% female Race/ethnicity: 77% Non-Hispanic White BMI (m ± SD): 30.14 (8.33)	Individualized mHealth 6 months Fitbit, study website, text messages, moderated Facebook group Self-help comparison (Fitbit, unmoderated Facebook group) Assessments at baseline, 3, 6, and 12 months	MVPA Light PA Steps Sedentary behaviors	Theory: SCT and SDT BCTs: I:48 C: 26 • Retention was 90% at 6 months • Promising rating: promising
**Wurz** [51] (2020), Canada To evaluate the effects of a 12-week PA intervention	16 AYAS ages 15–39, < 5 years post-treatment, sedentary lifestyle I: *n* = 7 C: waitlist control (*n* = 9) Age (m ± SD): 32.84 (7.93) Gender: 87.5% female Race/ethnicity: NR BMI (m ± SD): 31.86 (8.16)	Individualized Supervised PA program with aerobic and strength training 4x/week (2 supervised sessions in weeks 1–6) 12 weeks Assessments at baseline, 6, and 12 weeks	Feasibility Acceptability MVPA BMI Physical function Self-efficacy Self-esteem	Theory: EXSEM BCTs: 21 • Change in PA not reported • 94% retention at 12 weeks • Promising rating: non-promising

*N* = 14 studies with 15 intervention arms were included in the systematic review and analysis of BCTs. Li et al. (2023) included two intervention arms compared to a control condition: (1) PA intervention and (2) PA + behavioral activation (BA), *I* = Intervention, *C* = Control, *NR* = Not reported

### Study characteristics

The primary objective for most studies was feasibility and acceptability (*N* = 8) [42–48, 51], and all studies had multiple behavioral and psychosocial outcomes of interest. All interventions focused on improving PA (*N* = 15) [42–55], and only two studies included dietary components [47]. Most studies included two-arm intervention-control comparisons; Li et al. compared two intervention groups to one control [55]. Keadle et al. compared two PA intervention groups: PA or PA plus a charity donation incentive if step goals were achieved [43]. For the meta-analysis, the intervention group testing the additional novel component was considered the intervention group. Of the remaining thirteen studies, seven compared an intervention to standard care (54%), three utilized a waitlist control, and three had a self-help control group that included the provision of a Fitbit activity tracker.

The median length of follow-up was 3 months, with a range of 10 weeks to 12 months. Most interventions were delivered remotely (*N* = 11) [42–50, 53, 55] via mHealth (*N* = 7) [42–45, 48, 49, 55], consisting of a combination of Fitbits (*N* = 5) [42, 43, 48, 49, 55], websites (*N* = 4) [44, 45, 48, 49], social media (*N* = 4) [42, 48, 49, 55], and text messages (*N* = 2) [47, 49] or telephone [53]. One study examined the effectiveness of a digital tool designed to help care teams deliver health behavior counseling and community resources. One study consisted primarily of mailed print materials [56], one was delivered via telephone [46], and one was delivered exclusively via text messages [47]. Three [51, 54] studies were individualized, supervised exercise programs with accredited exercise physiologists [52]. Thirteen studies cited health behavior theories as informing the study, most commonly Social Cognitive Theory (*N* = 7 [43, 45–49, 55]), Self-determination Theory (*N* = 4 [42–44, 49]), Transtheoretical Model (*N* = 3 [45, 46, 53]), and the Theory of Planned Behavior (*N* = 2) [50, 55].

#### Risk of bias

**Fig. 2:**
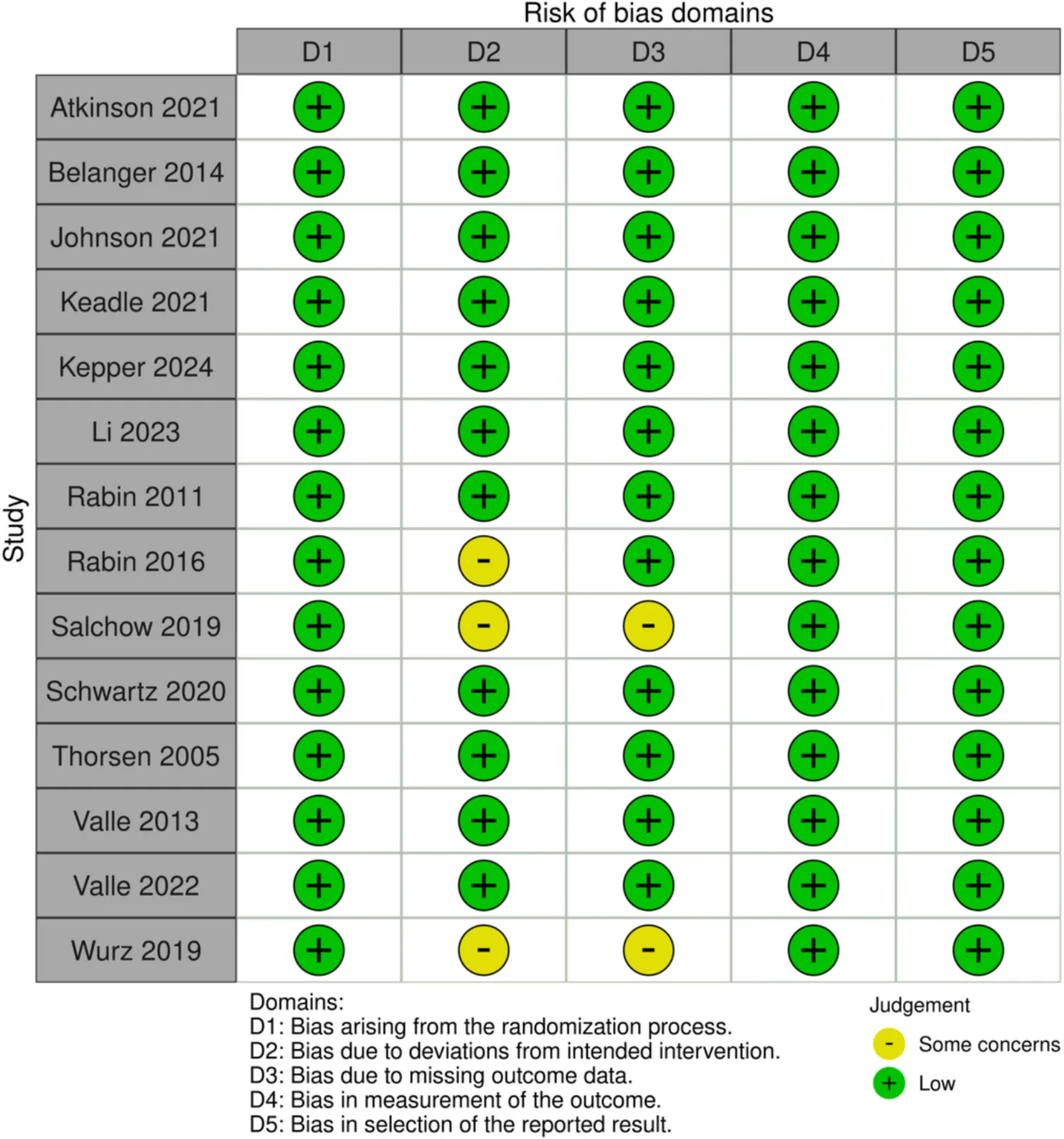
Risk of bias

Figure 2 presents details of the risk of bias assessments for individual studies [57, 58]. Most studies (*N* = 12, 80%) were judged to have a low risk of bias, and three were deemed to have some concerns. The main reasons for concern were differential attrition and incomplete outcome data not being analyzed using intent-to-treat methods.

#### Behavior change techniques (BCTs)

Of the fourteen studies (fifteen intervention arms), eight (57% [43, 44, 46–48, 52, 54, 55]) were judged to be promising, four (29% [45, 49, 50, 53]) were quite promising, and two (14% [42, 51]) were non-promising (Table 2). Fifty-seven BCTs were observed in at least one intervention. All study intervention arms (*N* = 15) had identifiable BCTs present (mean, 23.3; range, 11–48 (Table 3)). BCTs present in all studies were goal setting (behavior and outcome), instruction on how to perform a behavior, information about health consequences, and delivered by a credible source. Other frequently utilized BCTs included social support (*N* = 14), behavioral practice (*N* = 13) and habit formation (*N* = 13), action planning (*N* = 12), self-monitoring of behavior and outcomes (*N* = 12), graded tasks (*N* = 11), and social reward (*N* = 11). BCTs observed in control arms ranged from 0 to 26 with an average of 8.4, most frequently information about health consequences (*N* = 7), credible source (*N* = 7), goal-setting (behavior (*N* = 5) and outcome (*N* = 6)), and self-monitoring of behavior (*N* = 6). The average number of BCTs implemented in promising interventions was 22.9 (SD 8.1), quite promising was 23.8 (SD 16.8), and non-promising was 24.5 (SD. 5.0). Across the fifteen intervention arms, the highest possible promising ratio for individual BCTs was 11 (action planning), and the lowest was 1 (social comparison, habit reversal, generalization of target behavior, comparative imagining of future outcomes, self-incentive, incentive (outcome), body changes, identification of self as role model, framing/reframing, valued self-identity, mental rehearsal of successful performance, vicarious consequences. Thirty-seven BCTs were not identified in any intervention descriptions. Twenty-seven BCTs with a promising ratio of ≥ 2 were included as potential moderators in univariate meta-regressions.

**Table 2:** Behavior change techniques identified in 14 studies of PA and dietary interventions among AYA cancer survivors

	Intervention arms (*N* = 15)^*^	Control (*N* = 11)	Very promising (*N* = 9)	Quite promising (*N* = 4)	Non-promising (*N* = 2)	All (*N* = 15)	Promise ratio/score
**Total number of techniques:** mean (SD), range	23.3 (7.9), 10–48	8.4 (9.1), 0–26	22.9 (8.1), 10–48	23.8 (16.8), 11–48	24.5 (5.0), 21.28		
**Behavior change technique**	***n* (%)**	***n* (%)**	***n***	***n***	***n***	***n***	
1.1 Goal Setting (behavior)	**15** (93)	**5** (45)	9	4	2	14	6.5
1.2 Problem Solving	**9** (60)	**2** (18)	7	2	-	9	9
1.3 Goal Setting (outcome)	**15** (100)	**6** (55)	9	4	2	15	6.5
1.4 Action planning	**12** (80)	**4** (36)	7	4	1	12	11
1.5 Review Behavior goal(s)	**11** (73)	**-**	7	2	2	11	4.5
1.6 Discrepancy between current behavior and goal	**5** (33)	**-**	4	1	0	5	5
1.7 Review outcome goal(s)	**2** (13)	**-**	1	1	-	2	2
2.2 Feedback on behavior	**10** (67)	**3** (27)	5	3	2	10	4
2.3 Self-monitoring of behavior	**12** (80)	**6** (55)	6	4	2	12	5
2.4 Self-monitoring of outcome(s) of behavior	**12** (80)	**4** (36)	6	4	2	12	5
2.6 Biofeedback	**5** (33)	**-**	4	-	1	5	4
2.7 Feedback on outcome(s) of behavior	**10** (67)	**3** (27)	7	2	1	10	9
3.1 Social support (unspecified)	**14** (93)	**4** (36)	9	3	2	14	6
3.2 Social support (practical)	**7** (47)	**2** (18)	5	1	1	7	6
3.3 Social support (emotional)	**6** (40)	**2** (18)	4	1	1	6	5
4.1 Instruction on how to perform a behavior	**15** (100)	**6** (55)	9	4	2	15	6.5
5.1 Information about health consequences	**15** (100)	**7** (64)	9	4	2	15	6.5
5.3 Information about social and environmental consequences	**3** (20)	**1** (9)	2	1	-	3	3
5.4 Monitoring of emotional consequences	**4** (27)	**-**	3	1	-	4	4
5.6 Information about emotional consequences	**8** (53)	**3** (27)	5	2	1	8	7
6.1 Demonstration of the behavior	**8** (53)	**2** (18)	5	2	1	8	7
6.2 Social comparison	**2** (13)	**1** (9)	-	1	1	2	1
7.1 Prompts/cues	**9** (60)	**4** (36)	6	2	1	9	8
8.1 Behavioral practice/rehearsal	**13** (87)	**2** (18)	7	3	2	13	5.5
8.2 Behavior substitution	**2** (13)	**1** (9)	1	1	-	2	2
8.3 Habit formation	**13** (87)	**1** (9)	8	3	2	13	5.5
8.4 Habit reversal	**1** (7)	**1** (9)	-	1	-	1	1
8.6 Generalization of target behavior	**1** (7)	**-**	1	-	-	1	1
8.7 Graded tasks	**11** (73)	**1** (9)	8	1	2	11	4.5
9.1 Credible source	**15** (100)	**7** (64)	9	4	2	15	6.5
9.2 Pros and cons	**2** (13)	**-**	1	1	-	2	2
9.3 Comparative imagining of future outcomes	**1** (7)	**-**	-	1	-	1	1
10.1 Material incentive (behavior)	**2** (13)	**-**	1	1	-	2	2
10.2 Material reward (behavior)	**2** (13)	**-**	1	1	-	2	2
10.3 Non-specific reward	**1** (7)	**-**	-	-	1	1	-
10.4 Social reward	**11** (73)	**1** (9)	7	2	2	11	4.5
10.5 Social incentive	**3** (20)	**-**	1	1	1	3	2
10.6 Non-specific incentive	**1** (7)	**-**	-	-	1	1	-
10.7 Self-incentive	**1** (7)	**-**	-	1	-	1	1
10.8 Incentive (outcome)	**2** (13)	**-**	1	-	1	2	1
10.9 Self-reward	**3** (20)	**1** (9)	2	1	-	3	3
10.10 Reward (outcome)	**6** (40)	**1** (9)	4	1	1	6	5
11.2 Reduce negative emotions	**4** (27)	**2** (18)	3	1	-	4	4
12.1 Restructuring the physical environment	**8** (53)	**4** (36)	5	1	2	8	3
12.2 Restructuring the social environment	**6** (40)	**-**	3	2	1	6	5
12.5 Adding objects to the environment	**9** (60)	**4** (36)	6	1	2	9	3.5
12.6 Body changes	**2** (13)	**-**	1	-	1	2	1
13.1 Identification of self as role model	**1** (7)	**-**	-	1	-	1	1
13.2 Framing/reframing	**1** (7)	**-**	-	1	-	1	1
13.4 Valued self-identity	**1** (7)	**-**	-	1	-	1	1
13.5 Identity associated with changed behavior	**1** (7)	**-**	-	1	-	1	1
15.1 Verbal persuasion about capability	**8** (53)	**-**	4	3	1	8	7
15.2 Mental rehearsal of successful performance	**1** (7)	**-**	-	1	-	1	1
15.3 Focus on past success	**2** (13)	**-**	-	2	-	2	2
15.4 Self-talk	**3** (20)	**-**	1	2	-	3	3
16.2 Imaginary reward	**2** (13)	**-**	-	2	-	2	2
16.3 Vicarious consequences	**1** (7)	**-**	1	-	-	1	1

*N* = 14 studies with 15 intervention arms were included in the systematic review and analysis of BCTs

**Table 3:** Univariate meta-regression analyses for selected study characteristics

Moderator variable	*n*	*k*	*g*	SE	95% CI	*p-value*	*F-test*	*Q*_b_	*σ*^2^_Level3_	*σ*^2^_Level2_
**Overall effect of PA**	13	44	0.378	0.09	0.183, 0.573	0.002		138.84***	0.039	0.055
**Types of PA**							7.9 (8, 4)*	114.35***	0.033	0.065
Light PA	3	4	0.217	0.16	− 0.399, 0.826	0.30				
Moderate PA	5	5	0.448	0.20	− 0.066, 0.961	0.07				
Vigorous PA	5	5	0.216	0.10	− 0.050, 0.482	0.08				
MVPA	11	12	0.331	0.09	0.139, 0.524	< 0.01				
Total PA	7	7	0.421	0.14	0.069, 0.774	0.03				
VO^2peak^	2	2	0.548	0.04	0.123, 0.974	0.04				
Sedentary behavior	3	4	0.460	0.31	− 0.749, 1.670	0.26				
Steps	2	2	0.465	0.10	− 0.400, 1.327	0.10				
Walking (min)	3	3	0.532	0.28	− 0.437, 1.502	0.16				
**Measurement type**							0.06 (1, 11)	138.43***	0.039	0.050
Objective measures	5	12	0.389	0.07	0.186, 0.591	< 0.001				
Self-report Measures	10	32	0.375	0.10	0.143, 0.607	< 0.001				
**Control group type**							1.54 (2, 10)	133.36***	0.038	0.047
Standard care	7	23	0.457	0.14	0.104, 0.809	0.02				
Waitlist	2	4	0.522	0.50	− 5.878, 6.913	0.49				
Self-help	4	17	0.220	0.03	0.102, 0.337	0.01				
**Theory-based**							1.41 (1, 11)	137.41***	0.041	0.048
Yes	11	41	0.494	0.05	− 0.106, 1.090	0.06				
No	2	3	0.364	0.10	0.138, 0.590	< 0.01				
**Theories used (#)**							12.1 (2, 10)**	131.18***	0.029	0.045
0	2	3	0.494	0.05	− 0.105, 1.094	0.06				
1	5	19	0.150	0.05	− 0.004, 0.304	0.05				
2	6	22	0.548	0.18	0.024, 1.071	0.04				
**Theory of planned behavior**							1.96 (1, 11)	133.78***	0.017	0.049
Yes	3	13	0.567	0.20	− 0.289, 1.423	0.10				
No	10	31	0.273	0.07	0.100, 0.446	< 0.01				
**Self-determination theory**							3.13 (1, 11)	134.22***	0.034	0.046
Yes	4	17	0.225	0.03	0.103, 0.348	0.01				
No	9	27	0.455	0.13	0.156, 0.754	< 0.01				
**Social cognitive theory**							2.4 (1, 11)	136.70***	0.035	0.047
Yes	7	25	0.494	0.15	0.108, 0.879	0.02				
No	6	19	0.236	0.08	0.021, 0.450	0.04				
**Trans-theoretical model**							0.002 (1, 11)	138.69***	0.043	0.048
Yes	3	9	0.362	0.45	− 2.180, 2.903	0.53				
No	10	36	0.384	0.09	0.187, 0.581	< 0.01				

Effect sizes explain the change in PA from baseline to follow-up in favor of the intervention group

*N* number of intervention groups contributing effect sizes, *k* number of effect sizes, *g* Hedges’ g, *SE* standard error, *CI* confidence interval, *Q*_b_ measure of heterogeneity, *σ*^2^_Level3_ between-study variance, *σ*^2^_Level2_ within-study variance

^*^p < 0.05; **p < 0.01; ***p < 0.001

#### Dietary outcomes

Due to the study designs and outcome measures, dietary interventions could not be meta-analyzed. Kepper et al. evaluated the preliminary efficacy of a digital tool used by healthcare teams to counsel AYAs to improve PA and food intake behaviors compared with a waitlist control [44]. At baseline, 68% of all AYAs (*N* = 55) reported meeting PA recommendations and, on average, met 1.5 (of 5) food intake recommendations (whole grains, fruits, vegetables, high-sugar snacks, sugar-sweetened beverages). At the 3-month follow-up, intervention participants improved their food intake recommendations that they met to 2.1, but this was not significantly different from the control group. Both groups increased their willingness to change PA and dietary behaviors, and intervention participants significantly increased their self-efficacy for PA and healthy food intake compared with the waitlist control. Schwartz et al. [47] tested a text message intervention designed to improve physical and psychosocial well-being among 31 AYAs compared with standard care among 30 AYAs. All participants selected one of six health-related goals: healthy eating, smoking cessation, re-engaging in school, re-engaging in social activities, increasing PA, and improving sleep/fatigue; 36% chose to increase PA, and 34.4% chose healthy eating. Of intervention participants who chose healthy eating, 29% improved their fruit and vegetable intake (vs. 15% in standard care, *p* = 0.049), whole grain intake (31%, ns), and processed foods (39%, ns). 71% of intervention participants improved health knowledge.

#### Meta-analysis and sensitivity analyses

##### Overall effect

**Fig. 3:**
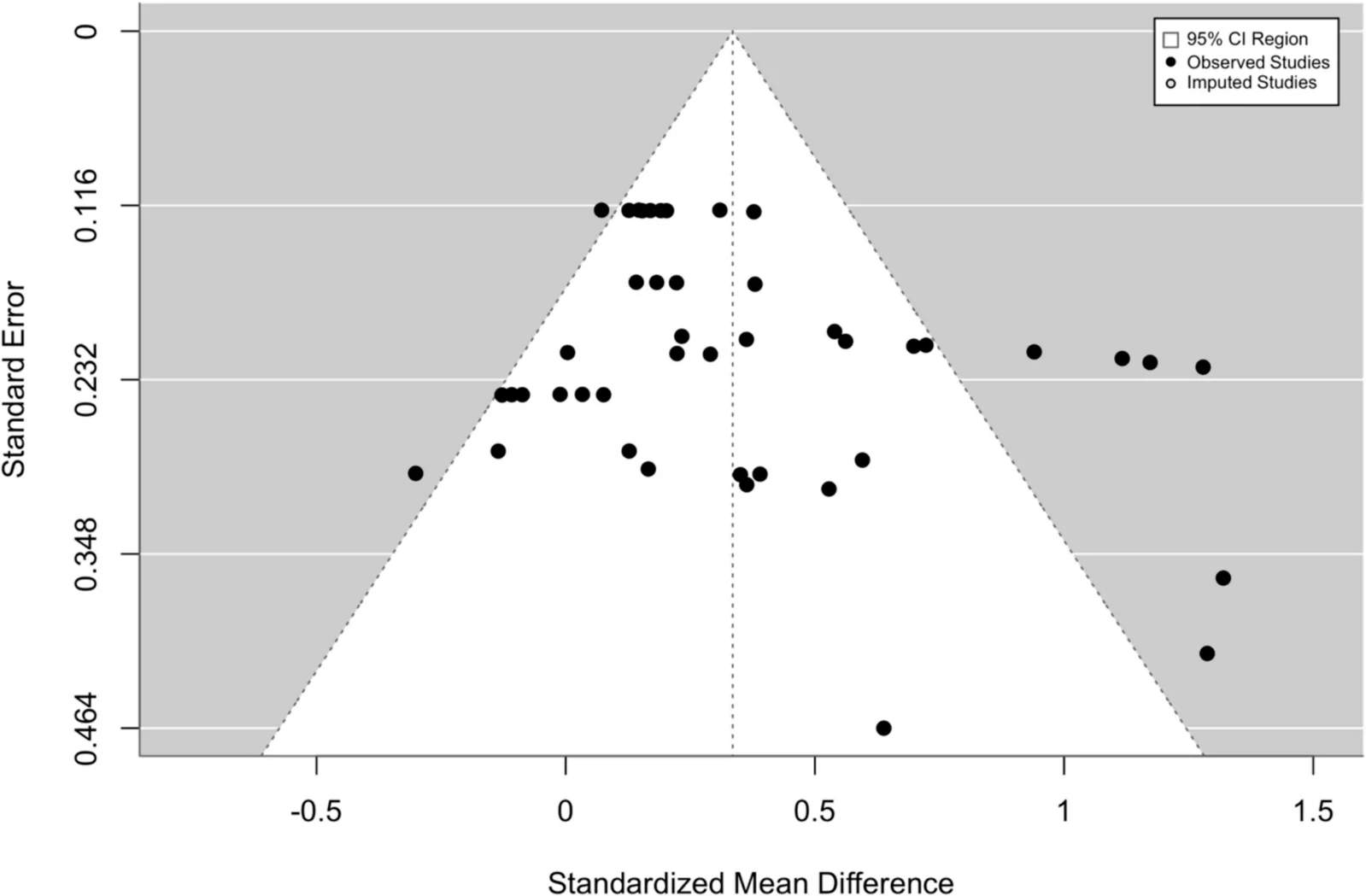

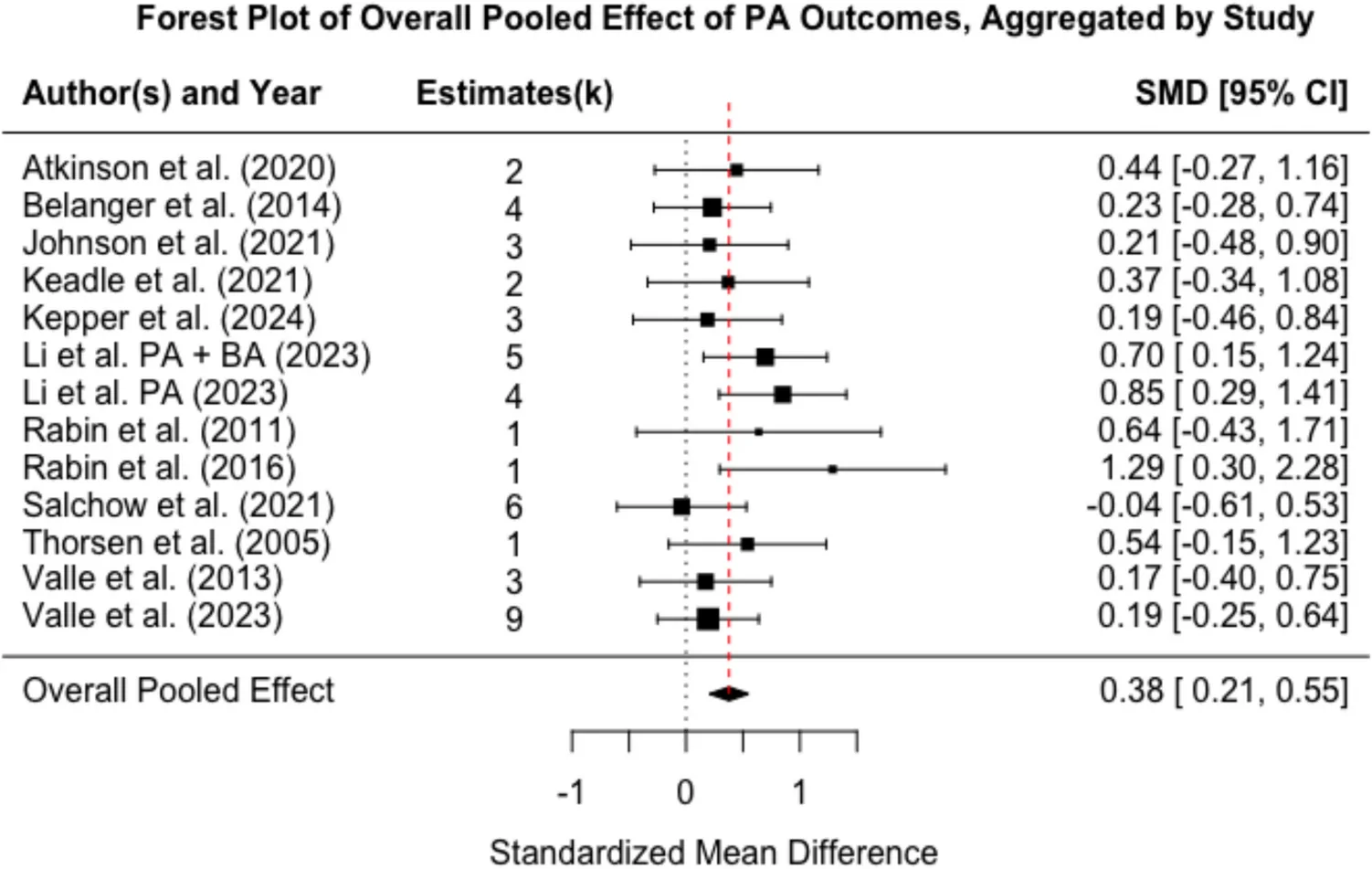
**A**. Forest plot of meta-analysis of PA interventions (*N* = 13). **B**. Funnel plot of PA outcomes

One outlier was identified and removed. The multilevel meta-analysis consisted of 13 PA interventions, with 44 effect sizes, resulting in an overall effect size of 0.378 (95% CI, 0.183–0.573; *p* < 0.01). The results indicate that behavior change interventions had a small to moderate significant effect on PA among AYA cancer survivors from baseline to follow-up compared to control conditions (Fig. 3). The estimated variance components were *σ*^2^_Level3_ = 0.039 and *σ*^2^_Level2_ = 0.055. This means that *I*^2^_Level3_ = 30.2% of the total variation can be attributed to between-study heterogeneity, and *I*^2^_Level2_ = 42.9% to within-study heterogeneity. We found that the three-level model provided a significantly better fit than a two-level model with the level three heterogeneity constrained to zero (*χ*^2^ = 18.07; *p* < 0.001). Orwin’s method to calculate fail-safe *N* indicated that 269 studies with non-significant findings would need to exist to reduce the effect size to a trivial effect size of *g* = 0.05. Inspection of the funnel plots of these effects was symmetrical (*p* = 0.07), and the trim and fill analysis to correct for publication bias did not impute any effect sizes, suggesting that publication bias is not likely to influence our result (Table 4) [40]. Differential attrition, and complete outcome data being analyzed instead of using intent-to-treat methods, may bias these results.

**Table 4:** Results from univariate meta-regression analyses of individual behavior change techniques on overall change in PA effect size

Behavior Change Technique		n	*k*	*g*	SE	95% CI	*p-value*	*F-test*	*σ*^2^_Level3_	*σ*^2^_Level2_
1.2 Problem solving	Present	11	39	0.391	0.13	0.080, 0.703	0.02	0.042 (1, 11)	0.045	0.048
	Absent	8	33	0.360	0.08	0.123, 0.598	0.02			
1.4 Action planning	Present	11	39	0.389	0.10	0.160, 0.618	< 0.01	0.231 (1, 11)	0.044	0.047
	Absent	2	5	0.321	0.10	− 0.932, 1.575	0.19			
1.5 Review behavior goal(s)	Present	10	37	0.394	0.11	0.143, 0.644	< 0.01	0.195 (1, 11)	0.045	0.048
	Absent	3	7	0.330	0.10	− 0.220, 0.880	0.11			
1.6 Discrepancy between current behavior and goal	Present	5	24	0.415	0.15	− 0.006, 0.835	0.05	0.139 (1, 11)	0.043	0.048
	Absent	8	20	0.347	0.11	0.080, 0.613	0.02			
2.2 Feedback on behavior	Present	9	35	0.369	0.11	0.107, 0.632	0.01	0.053 (1, 11)	0.046	0.047
	Absent	4	9	0.414	0.16	− 0.158, 0.987	0.10			
2.3 Self-monitoring of behavior	Present	11	39	0.392	0.10	0.162, 0.622	< 0.01	0.288 (1, 11)	0.044	0.048
	Absent	2	5	0.305	0.13	− 1.305, 1.915	0.25			
2.4 Self-monitoring of outcome(s) of behavior	Present	11	41	0.364	0.10	0.138, 0.590	< 0.01	1.41 (1, 11)	0.041	0.048
	Absent	2	3	0.494	0.05	− 0.106, 1.090	0.06			
2.6 Biofeedback	Present	9	32	0.682	0.08	0.394, 0.970	< 0.01	25.1 (1, 11)***	0.000	0.045
	Absent	4	12	0.218	0.04	0.096, 0.339	< 0.01			
2.7 Feedback on outcome(s) of behavior	Present	9	30	0.458	0.12	0.176, 0.740	< 0.01	2.48 (1, 11)	0.035	0.047
	Absent	4	14	0.209	0.11	− 0.147, 0.566	0.15			
3.1 Social support (unspecified)	Present	12	40	0.400	0.10	0.180, 0.620	< 0.01	2.98 (1, 11)	0.046	0.047
	Absent	1	4	0.231	0.002	0.203, 0.259	< 0.01			
3.2 Social support (practical)	Present	6	25	0.470	0.15	0.055, 0.884	0.03	1.21 (1, 11)	0.038	0.048
	Absent	7	19	0.278	0.09	0.062, 0.495	0.02			
3.3 Social support (emotional)	Present	5	24	0.421	0.15	− 0.006, 0.847	0.05	0.186 (1, 11)	0.042	0.048
	Absent	8	20	0.342	0.11	0.078, 0.605	0.02			
5.4 Monitoring of emotional consequences	Present	4	19	0.578	0.23	− 0.269, 1.424	0.11	1.89 (1, 11)	0.034	0.046
	Absent	9	25	0.255	0.06	0.106, 0.404	< 0.01			
5.6 Information about emotional consequences	Present	8	33	0.402	0.13	0.086, 0.718	0.02	0.17 (1, 11)	0.045	0.048
	Absent	5	11	0.340	0.08	0.113, 0.566	0.02			
6.1 Demonstration of the behavior	Present	7	25	0.404	0.11	0.130, 0.677	0.01	0.070 (1, 11)	0.047	0.047
	Absent	6	19	0.351	0.17	− 0.103, 0.804	0.10			
7.1 Prompts/cues	Present	9	34	0.403	0.10	0.157, 0.649	< 0.01	0.198 (1, 11)	0.042	0.048
	Absent	4	10	0.307	0.19	− 0.367, 0.980	0.22			
8.1 Behavioral practice/rehearsal	Present	12	40	0.400	0.002	0.180, 0.620	< 0.01	2.98 (1, 11)	0.046	0.047
	Absent	1	4	0.231	0.10	0.203, 0.259	< 0.01			
8.3 Habit formation	Present	12	40	0.400	0.002	0.180, 0.620	< 0.01	2.98 (1, 11)	0.046	0.047
	Absent	1	4	0.231	0.10	0.203, 0.259	< 0.01			
8.7 Graded tasks	Present	10	30	0.430	0.11	0.168, 0.692	< 0.01	2.09 (1, 11)	0.035	0.048
	Absent	3	14	0.247	0.05	− 0.129, 0.623	0.09			
10.4 Social reward	Present	9	36	0.375	0.12	0.100, 0.651	0.01	0.021 (1, 11)	0.045	0.048
	Absent	4	8	0.398	0.11	0.013, 0.783	0.05			
10.5 Social incentive	Present	3	14	0.235	0.05	− 0.009, 0.479	0.05	2.49 (1, 11)	0.038	0.047
	Absent	10	30	0.431	0.11	0.166, 0.696	< 0.01			
10.10 Reward (outcome)	Present	6	26	0.425	0.13	0.071, 0.780	0.03	0.313 (1, 11)	0.040	0.048
	Absent	7	18	0.326	0.12	0.023, 0.630	0.04			
11.2 Reduce negative emotions	Present	4	19	0.578	0.23	− 0.269, 1.424	0.11	1.89 (1, 11)	0.034	0.046
	Absent	9	25	0.255	0.06	0.106, 0.404	< 0.01			
12.1 Restructuring the physical environment	Present	7	27	0.459	0.13	0.113, 0.804	0.02	1.39 (1, 11)	0.037	0.048
	Absent	6	17	0.265	0.10	0.005, 0.524	0.05			
12.2 Restructuring the social environment	Present	6	25	0.431	0.14	0.045, 0.818	0.04	0.36 (1, 11)	0.041	0.048
	Absent	7	19	0.324	0.11	0.047, 0.602	0.03			
12.5 Adding objects to the environment	Present	8	30	0.431	0.12	0.137, 0.725	0.01	0.79 (1, 11)	0.039	0.048
	Absent	5	14	0.281	0.12	− 0.079, 0.641	0.09			
15.1 Verbal persuasion about capability	Present	8	35	0.379	0.12	0.079, 0.678	0.02	0.002 (1, 11)	0.045	0.048
	Absent	5	9	0.386	0.09	0.127, 0.646	0.02			

*N* number of intervention groups contributing effect sizes, *k* number of effect sizes, *g* Hedges’ g, SE standard error, CI confidence interval, *σ*^2^_Level3_ between-study variance, *σ*^2^_Level2_ within-study variance

^*^p < 0.05; **p < 0.01; ***p < 0.001

#### Moderator analyses

To explore the substantial heterogeneity in the overall effect size, we used multilevel meta-regressions to examine the effects of nine moderators related to study characteristics (e.g., subtypes of PA, type of control group used, and theoretical underpinning), the total number of BCTs utilized (Table 4), and 27 BCTs (Table 5). The between-study *Q*-statistic (*Q*_b_) was used to evaluate heterogeneity for each univariate model. All *Q*_b_ values were significant for the study characteristics and BCT moderators examined.

### Study characteristics

**Fig. 4:**
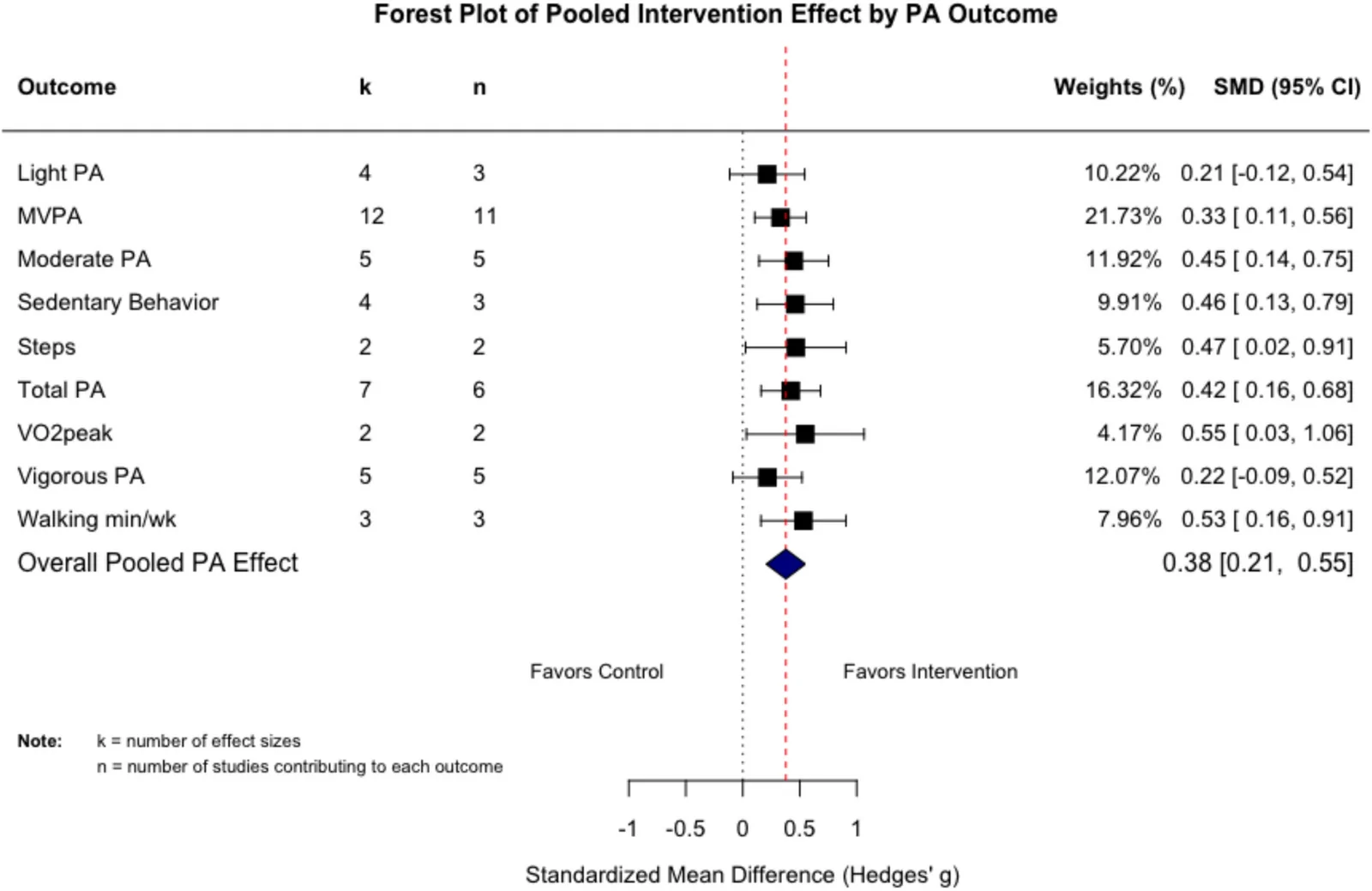
Forest plot of moderation analysis of PA outcome measure on the effect of intervention effect

The level or type of PA outcome assessed was a statistically significant moderator of the overall intervention effect (*F*_(8,4)_ = 7.9, *p* < 0.001) (Fig. 4). The effect was slightly attenuated for MVPA (*g* = 0.331, *k* = 12, 95% CI, 0.139–0.524, *p* < 0.01), and somewhat larger for total PA (*g* = 0.421, *k* = 7, 95% CI, 0.069–0.774, *p* = 0.03), and the largest effect was observed in the two studies that measured VO^2peak^ (*g* = 0.548, *k* = 2, 95% CI, 0.123–0.974, *p* = 0.04). We found statistically significant differences based on the number of behavior change theories used (*F*(_2,10)_ = 12.1, *p* < 0.01). Studies that used two theories (*N* = 6) had a larger moderate effect size (*g* = 0.548, *k* = 22, 95% CI, 0.024–1.071, *p* = 0.04). No other moderator analyses of study characteristics were significant.

### Behavior change theories (BCTs)

Moderator analyses were conducted on 27 individual BCTs (Table 4). There were significant differences in interventions that used BCT 2.6 “biofeedback,” “providing feedback about the body (e.g., physiological or biochemical state) using an external monitoring device as part of a behavior change strategy,” most commonly heart rate trackers to determine exercise intensity (*F*_(1,11)_ = 25.1, *p* < 0.001). Interventions that utilized biofeedback had a moderate to large effect (*g* = 0.682, *N* = 5, 95% CI, 0.394–0.970, *p* < 0.001), compared to a small effect in those studies that did not (*g* = 0.218, *N* = 10, 95% CI, 0.096–0.339, *p* < 0.001). Although effect sizes were larger in the presence of several BCTs (vs. absence), no other between-level comparisons were significant. Other BCTs with large differences in effect size between studies that did and studies that did not utilize it were “feedback on behavior,” “social support (practical),” “monitoring of emotional consequences,” and “reduce negative emotions.” None of the moderators examined reduced the unexplained effect heterogeneity to a non-significant level.

## Discussion

This systematic review and meta-analysis characterized and evaluated the effects of interventions targeting PA and dietary behaviors among post-treatment AYA cancer survivors. This work synthesized the effects of 15 studies with 1,169 post-treatment AYA cancer survivors, and meta-analyzed the effects of 13 intervention arms compared to a control, representing 44 effect sizes across 9 measures of PA. Most studies (80%) were deemed to have a low risk of bias. We found that interventions had a moderate effect on PA behavior at post-intervention follow-up compared with control groups (*g* = 0.38, *p* = 0.002). The effect was slightly attenuated for MVPA (*g* = 0.33, *p* < 0.01) and larger in magnitude for total PA (*g* = 0.42, *p* = 0.03). The largest effect size for subtypes of PA was observed for VO2peak (*g* = 0.548, *p* = 0.04), a measure of cardiorespiratory fitness. The results show an overall significant positive effect on PA, similar in the magnitude of effect sizes of other meta-analyses for PA in cancer survivors that reported effect sizes of 0.25–0.33 [32, 59, 60]. Our results for total PA (0.38) are comparable (0.35) to those reported in another recent review that focused primarily on young cancer survivors, including participants ages 3–18, and focused on interventions during treatment [20].

Exploratory moderator analyses indicated that measurement type (objective vs. self-report), type of control group, and theoretical basis did not distinguish effective PA interventions. However, there were significant within-group differences for both the control group choice and the use of behavior change theories. The effect sizes were larger for studies that compared to standard care (*g* = 0.46, *p* = 0.02) and were smaller for studies that had a “self-help” control group (*g* = 0.22, *p* = 0.01). This finding demonstrates that the standardized mean difference may not adequately capture an intervention’s effect because both groups (i.e., intervention and comparator) may have increased their PA. Additionally, four studies utilized wearable activity trackers in both the intervention and control groups. Johnson et al. [42] provided Fitbits to the control group without further instruction. Valle et al. utilized a self-help control group that received a Fitbit and a private unmoderated Facebook group with study participants [42, 48, 61]. Keadle et al. compared two active intervention groups that received the same core intervention consisting of a Fitbit, step goal, and weekly electronic newsletter [43], with one group receiving the added incentive of charity donations if their step goal was achieved. Li et al. also had two PA intervention arms that received the same core intervention of an exercise manual, PA tracker, and private chat group, compared to a standard control group [55]. In these studies, intervention and control conditions increased their PA, which may contribute to the between-study heterogeneity by attenuating the effect size.

The advantage of standardized effect sizes is that they allow for comparisons across different populations and study designs in meta-analysis; however, standardization separates the effect size from the original metrics, making real-world interpretations difficult. Wright et al. highlight an alternative to interpreting effect sizes in terms of small, medium, and large by using benchmarking, which compares the effectiveness of any particular study to the effectiveness of previous studies in that domain [62]. Using this approach, they benchmarked the effectiveness of interventions to promote PA and reported percentile values for intervention effect sizes and indices of PA across samples, settings, measures, and follow-up periods [63]. Based on this metric, our observed effect size of 0.22 for comparisons using active “self-help” control groups is below the 5th percentile of effect sizes in cancer survivors, which equates to an increase of 20.8 min/day of MVPA, 1761 steps per day, and 5.7% more meeting guideline recommendations.

Having a theoretical basis (yes/no) did not moderate the effect of PA interventions; however, interventions that used any behavior change theory had a larger effect (*g* = 0.494, *p* = 0.06) than those that did not (*g* = 0.364, *p* < 0.01). The number of theories used in the intervention design did have a moderator effect; studies that used two behavior change theories produced larger effect sizes (*g* = 0.55, *p* = 0.04) compared to studies that mentioned the use of one or zero theories. The theories associated with more effective interventions were the Theory of Planned Behavior (TPB) and the Social Cognitive Theory (SCT). SCT was the most frequently used theory in our analysis. These mixed results regarding the efficacy of theory align with what has been reported in previous reviews [33, 59, 60]. A possible explanation may be that it depends on the authors explicitly stating what theories they used and how they operationalized them in the intervention. Pre-registration of protocols allows for interventions to be described in greater detail, including conceptual models that explain how a theory is being implemented, what theoretical constructs are being targeted, and how they are being evaluated.

Using BCTs to describe interventions, we hoped to explain some of the heterogeneity in PA outcomes and identify “key ingredients” of behavior change interventions for AYA cancer survivors. The number of BCTs used in intervention groups and the most promising BCTs examined were not associated with increased effectiveness. A caveat to this finding is the identification of “core” BCTs that were identified in all intervention arms; these include goal setting (behavior and outcome), instruction on how to perform the behavior, information about health consequences, and delivery from a credible source. Most interventions also utilized self-monitoring, social support, behavioral practice, habit formation, and graded tasks—BCTs which are frequently used in behavioral interventions and have consistently been associated with improving PA [41, 64]. The majority of interventions also used wearable activity trackers, which are captured by the BCTs, “adding objects to the environment”(*N* = 9) and “restructuring the physical environment” (*N* = 8) [28]. Along with a theoretical basis, BCTs should be identified a priori*,* and how they will be implemented and evaluated to enable transparency, replication, and strengthen the causal evidence linking theory and behavior change to continue building the most effective interventions. There is also a need to develop interventions that can sustain behavior change, and future research should investigate BCTs associated with the maintenance of effects.

The impact on intervention effect was mixed among the individual BCTs examined in univariate regression models. The only BCT that resulted in a statistical difference in effect sizes was “biofeedback” (*g* = 0.68, *p* < 0.01 vs. *g* = 0.22, *p* < 0.01). However, there were several additional BCTs that had larger effect sizes when present, although not statistically different. BCTs that were positively associated with intervention effect were feedback on outcomes of behavior, all types of social support (practical, emotional, unspecified), information about and monitoring of emotional consequences, reducing negative emotions, prompts/cues, behavioral practice, habit formation, graded tasks, restructuring the physical environment, and adding objects to the environment. BCTs associated with reduced effect sizes included feedback on behavior and self-monitoring of behavior outcomes. Regression models only examined BCTs in the intervention groups and did not compare between intervention and control groups or compare “very promising” to “quite promising” or “non-promising” interventions. BCTs were similar across promise rating categories and suggest that there is more nuance to study design, context, and delivery that may contribute to effectiveness. The two interventions that included a dietary component could not be meta-analyzed but were part of multi-component “promising” interventions, meaning significant between-group effects from baseline to follow-up. These provide the first evidence for changing dietary behaviors in AYAs and demonstrate promising preliminary findings. The paucity of studies in this area highlights the need for dietary interventions for AYAs.

### Strengths and limitations

To our knowledge, this is the first meta-analysis to review the efficacy of behavioral interventions in improving PA and dietary outcomes among post-treatment AYA cancer survivors. This was also the first review to characterize BCTs of interventions for AYAs to elucidate some of the findings. We identified a set of “core” BCTs in all intervention arms as well as BCTs that are frequently used in behavioral interventions and have consistently been shown to be associated with improving PA [41, 64].

Additionally, several limitations should be acknowledged. The existing literature consists of small sample sizes with predominantly non-Hispanic White female participants, which may limit the generalizability of the findings to other races and ethnicities, males, or individuals undergoing active cancer treatment. Due to small sample sizes and the number of studies, the meta-regressions are exploratory, and larger, more diverse samples are needed to explore the impact of BCTs on intervention effects. Most of the studies evaluated were pilot or feasibility studies, with a median length of 3 months; longer follow-up periods are needed to assess the maintenance of behavior change. The nature of behavioral interventions makes it impossible to blind participants and, in most cases, interventionists, potentially impacting the quality of risk of bias assessments. Most studies utilized self-report methods to assess PA, which can lead to the overestimation of MVPA and underestimation of sedentary behaviors, so future studies should also incorporate objective measures to strengthen the findings [65]. Despite our comprehensive efforts, we may have missed some relevant studies during the literature search, particularly those that did not meet specific search criteria.

Furthermore, to characterize interventions by the BCTs they used, we retrospectively coded BCTs from descriptions of the existing interventions. Valle et al. [61] was the only study to prospectively code their intervention components and the intended BCTs targeting behavior change. This retrospective method depends on how explicitly and thoroughly an intervention is described, which may be insufficient. To evaluate the potential efficacy of BCTs identified in interventions, a “promise ratio” was used to characterize the impact of individual BCTs on improving participants’ health behaviors. Each study was characterized according to its potential to improve health behavior outcomes post-intervention, and each BCT was given a “promise ratio” based on the times it was utilized in promising vs non-promising studies. Promise ratios were not calculated for BCTs included in non-promising interventions only. An individual BCT had to have a promise ratio of ≥ 2 to be considered promising, with higher ratios indicating a more promising intervention. A limitation of using meta-regression is that reducing interventions to an effect size may result in unmeasured confounding that accounts for the associations observed. Another limitation of analyzing BCTs present in only effective interventions is that it may identify ineffective BCTs that are present and does not account for the interaction between BCTs [66]. Interventions may also vary in how a BCT is operationalized, implemented, and emphasized within the context of their intervention. BCTs may have been planned in the design of an intervention but not delivered to participants. Despite these limitations, this study is an important step in determining which BCTs have been used to improve PA behaviors among AYAs. It illustrates the level of detail needed to describe and evaluate future interventions.

## Conclusions

The findings of our systematic review and meta-analysis demonstrate that behavioral interventions were effective at increasing PA in AYA cancer survivors. Given the heterogeneity found in subgroup analyses, our results highlight the need for detailed reporting of intervention design and delivery, including how theory was operationalized, prospectively identifying how BCTs are planned to be implemented and measured, and examining their effectiveness post-intervention. This information can facilitate future evaluations of intervention effectiveness across studies and identification of effective intervention components. To better understand individual responses to behavioral interventions, future interventions should seek to include representative samples with diversity in age, gender, race, and ethnicity. Objective measures for PA and longer study durations or follow-up periods are needed to examine the maintenance of behaviors. The systematic review indicates a need for interventions that include dietary behaviors in addition to PA for AYAs.

## Data Availability

No datasets were generated or analysed during the current study.
